# A Malignant Glomus Tumor of the Liver Harboring MIR143-NOTCH2 Rearrangement: From Diagnosis to Management

**DOI:** 10.7759/cureus.30718

**Published:** 2022-10-26

**Authors:** Ruoyu Miao, Marilyn M Bui, Christine Walko, John E Mullinax, Andrew S Brohl

**Affiliations:** 1 Department of Hematology and Medical Oncology, Moffitt Cancer Center, Tampa, USA; 2 Department of Pathology, Moffitt Cancer Center, Tampa, USA; 3 Department of Individualized Cancer Management, Moffitt Cancer Center, Tampa, USA; 4 Department of Sarcoma, Moffitt Cancer Center, Tampa, USA

**Keywords:** chemotherapy, gene fusion, notch, next generation sequencing, malignant glomus tumor

## Abstract

A primary malignant glomus tumor of the liver is extremely rare and diagnostically challenging. We present an exceptional case of such with a diagnosis confirmed by *MIR143-NOTCH2* rearrangement. The case was successfully managed with neoadjuvant chemotherapy followed by surgery. This report highlights the utilization of molecular analysis to aid in the diagnosis of rare soft tissue malignancies and supports a multimodality approach to the treatment of large, high-grade malignant glomus tumors.

## Introduction

Glomus tumors are rare mesenchymal tumors arising from the modified smooth muscle cells of the glomus body [1-3]. They account for less than 2% of all soft tissue tumors [1,2]. Most glomus tumors are benign, slow-growing neoplasms that are commonly found in the distal extremities, particularly in the subungual layers of the digits. However, they can also be found in extracutaneous areas, such as the gastrointestinal tract, with the stomach being the most common site, mediastinum, lungs, kidney, and bone [1,4-7]. Primary malignant glomus tumors of the liver are extremely rare and diagnostically challenging. To date, only eight cases of glomus tumors in the liver have been reported in the literature [5,6]. Recently, NOTCH gene rearrangements have been identified in over half of glomus tumors, with *NOTCH2* being the most common abnormality and *CARMN* or *MIR143* as the most frequent gene fusion partner [2,3,8,9]. We present a case of malignant glomus tumor of the liver that was diagnosed with positive *MIR143-NOTCH2* rearrangement.

## Case presentation

**Figure 1:**
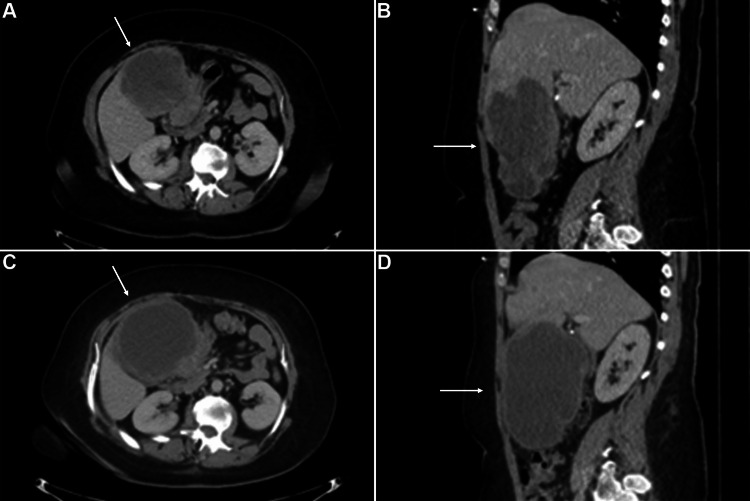
CT images of the liver lesion A, B) CT images (A axial, B sagittal) prior to chemotherapy demonstrated a 14.3 x 11.6 cm mass (arrows) originating from segment IVb of the liver. C, D) On repeat CT scan (C axial, D sagittal) after neoadjuvant chemotherapy, the tumor was more encapsulated with a notable increase in central tumor necrosis.

A 44-year-old woman with a past medical history significant only for gastroesophageal reflux disease presented to an emergency department with abdominal pain, early satiety, and low-grade fevers. A computed tomography (CT) scan demonstrated a 14.3 x 11.6 cm mass originating from segment IVb of the liver (Figures 1A, 1B).

The patient underwent a CT-guided biopsy that revealed a high-grade pleomorphic malignancy with extensive tumor necrosis and frequent mitotic activity. A pertinent immunohistochemical (IHC) panel was performed, and the tumor was positive for vimentin, but negative for markers of carcinoma (pancytokeratin AE1/AE3/CAM5.2, CDX2, HepPar, EMA, and synaptophysin), melanoma (S-100 protein, SOX10, and PRAME), hematopoietic malignancy (CD3, CD20, CD30, ALK1, and granzyme), mesothelioma (WT-1), and various sarcomas (CD34, ERG, SATB2, STAT6, DOG1, desmin, MyoD1, and NKX2.2). H3K27me3 was retained. The histomorphological and IHC features favored an undifferentiated pleomorphic sarcoma (UPS). Further immunohistochemical and molecular analysis was considered but deferred due to limited and highly necrotic biopsy sampling and the need to more urgently start therapy.

Further staging imaging with positron emission tomography (PET)/CT demonstrated a necrotic mass with a maximum SUV of 23.9 in segment IV of the liver extending to the gastric antrum and duodenum with no evidence of metastatic disease.

Given the working diagnosis of large, high-grade UPS, the patient was treated with three cycles of neoadjuvant chemotherapy consisting of doxorubicin, ifosfamide, and mesna. She tolerated the chemotherapy well and experienced an improvement in her pain and early satiety following therapy. Restaging CT scan after two cycles of chemotherapy demonstrated increased central necrosis of the tumor suggestive of treatment response (Figures 1C, 1D).

**Figure 2:**
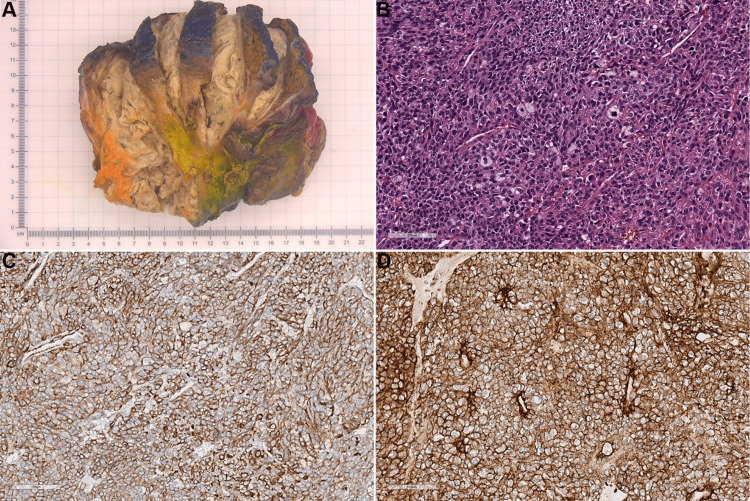
Pathological images of the malignant glomus tumor A) Gross image after formalin fixation shows the tumor is almost entirely necrotic (90%). B) The hemotoxylin-eosin stained histology slides show the tumor is composed of blue, round, epithelioid, and spindle cells arranged in an organoid pattern. There is prominent vasculature. Nuclear pleomorphism and mitotic activity are seen. C, D) The tumor cells are positive for actin and collagen IV.

Following recovery from chemotherapy, the patient underwent exploratory laparotomy with resection of the abdominal tumor, including en bloc antrectomy and segment IVb liver with Billroth II gastrojejunostomy reconstruction. The tumor was noted to be well-encapsulated clinically and pathologically. The gross specimen (Figure 2A) showed an 11x10 x 8 cm mass involving the liver parenchyma with 90% tumor necrosis. The histology (Figure 2B) demonstrated round, epithelioid, and spindle tumor cells arranged in an organoid pattern. There is a prominent vasculature. The immunostain workup showed the tumor cells were positive for synaptophysin but negative for cytokeratin AE1/AE3/CAM5.2, chromogranin, INSMS1, GATA3, inhibin, calretinin, c-MYC, desmin, MDM2, CDK4, CD117, DOG1, NXK2.2, WT1-1, ERG, CD99, S-100, SOX10, HMB45, and STAT6. SDHB was retained. The Ki-67 proliferation index was 20% by manual morphometric analysis. The EWSR1 and SS18 gene rearrangement testing by fluorescence in situ hybridization was also negative. Similar to the biopsy specimen, these largely negative findings did not support a diagnosis of neuroendocrine tumor, melanoma, dedifferentiated liposarcoma, gastrointestinal stromal tumor, angiosarcoma, Ewing sarcoma, desmoplastic small round cell tumor, solitary fibrous tumor, or adrenal tumor., etc, and therefore were suggestive of UPS. There was no angiolymphatic invasion. Surgical margins were free of tumors.

To help further establish the diagnosis, due to UPS being a diagnosis of exclusion, the resection specimen was subjected to molecular analysis. Molecular profiling was performed using a commercial targeted next-generation sequencing panel (FoundationOne CDx, Foundation Medicine, Inc., Cambridge, Massachusetts), which revealed a *MIR142-NOTCH2* fusion, along with *CDKN2A* loss (p16INK4a loss and p14ARF loss exons 2-3) and six variants of unknown significance (*ATM* G3029D, *AXIN1* V517I, *KDR* S1307G, *NOTCH2* R1911H, *RAD51B* H95R, *WHSC1* H528N). The *MIR143-NOTCH2* fusion involved a 3’ rearrangement breakpoint in intron 26 of *NOTCH2* and included exons 27-34 of *NOTCH2*. The fusion was reported in 389 read pairs. Additional IHC studies were performed showing the tumor cell was positive for smooth muscle actin (Figure 2C), collagen IV (Figure 2D), and smooth muscle myosin. The presence of *MIR143-NOTCH2* rearrangement, together with supportive IHC and morphological features, confirmed the diagnosis of a malignant glomus tumor.

The patient recovered well from surgery and is currently in active surveillance without evidence of disease ongoing at six months as of the time of this report.

## Discussion

Primary malignant glomus tumors of the liver are extremely rare and represent a challenge in diagnosis. We report a unique case of a primary glomus tumor of the liver that presented as a large, high-grade lesion with overtly malignant features. In this case, the undifferentiated cellular morphology and nonspecific immunohistochemistry posed a significant diagnostic challenge. Ultimately, molecular profiling with the discovery of *MIR143-NOTCH2* fusion helped establish the diagnosis.

The Notch signaling pathway is frequently dysregulated in human malignancy and NOTCH gene mutations have now been identified in a broad spectrum of cancers. In the past decade, NOTCH gene fusions have been reported in more than half of glomus tumors and are especially prevalent in malignant glomus tumors [2,3,8,9]. In the initial study including 33 glomus tumors by Mosquera et al., *NOTCH2* gene rearrangements were identified in 52% of glomus tumors, including all five malignant glomus tumors. Another 9% of glomus tumors were found to harbor NOTCH3 rearrangements. *MIR143, *a microRNA that has also been proposed to have tumor-suppressive effects, was detected as the gene fusion partner in two-thirds of the cases. In comparison, among other subtypes of the tumors of perivascular smooth muscle differentiation, only one out of 18 angioleiomyomas tested positive for *NOTCH2* gene rearrangement, myopericytoma, myofibroma, and myofibromatosis were negative [8]. Agaram et al. reported NOTCH gene fusions detected in 54% of glomus tumors, with *MIR143-NOTCH2* being the most common fusion (73%). NOTCH fusions were more frequently identified in malignant glomus tumors [3]. Long noncoding RNA *CARMN*, which is believed to have a role in the regulation of cellular differentiation, was annotated as the host gene of the *MIR143/145* cluster. In the study by Girard et al., the *CARMN-NOTCH2* translocation was identified in 88% of glomus tumors of the upper gastrointestinal tract and 33% of cutaneous glomus tumors [2]. Similarly, Papke et al. reported *NOTCH2* alterations in 80% of benign and malignant gastroesophageal glomus tumors and *CARMN-NOTCH2* was the most common translocation [9].

The pattern of *MIR143-NOTCH* translocations is reported to be highly conserved, in which the first exon of *MIR143* is fused to most of the NOTCH intracellular domain (NICD) [8]. It is speculated that the translocations remove the coding sequences for an extracellular NOTCH-negative regulatory region and preserve NOTCH intracellular sequences that are responsible for signaling, resulting in the activation of the NOTCH pathway and tumor development driven by the potent *MIR143* promoter [2,7,8]. NOTCH signaling represents an attractive target for a variety of cancers and has been actively explored for therapeutic options [10,11]. Most recently, Zhang et al. reported a durable response of LY3039478, an orally bioavailable γ-secretase inhibitor that cleaves NOTCH receptors within their transmembrane domains, allowing the NICD to be released from the membrane and translocate to the nucleus to form a transcription activation complex in a pediatric patient with metastatic malignant glomus tumor carrying a *CARMN-NOTCH1* fusion [7].

Although *BRAF* p.Val600Glu mutations have been reported in 6% of glomus tumors that present an opportunity for targeted therapy [12], this case did not harbor a *BRAF* mutation. In addition, typical glomus tumors are benign. However, malignant glomus tumors are aggressive; some large, visceral glomus tumors without overt malignant features also behaved aggressively [13]. There is scarce information in the literature regarding the behavior of the large malignant glomus tumor of the liver. Surgical resection with negative margins is the treatment of choice for glomus tumors of the liver, as for glomus tumors of other sites [5,6]. Given the rarity of the disease, little is known regarding the effectiveness of chemotherapy in malignant glomus tumors. Limited data consisting of isolated case reports of chemotherapy in patients with malignant glomus tumors have generally indicated poor response [14-16]. Due to the locally advanced presentation and initial working diagnosis of high-grade undifferentiated sarcoma, our patient was treated with neoadjuvant chemotherapy consisting of doxorubicin and ifosfamide, which resulted in clinical symptom improvement and suggestion of response by tumor necrosis both radiographically and pathologically. To our knowledge, this is the first reported case of neoadjuvant chemotherapy treatment for a malignant glomus tumor. The efficacy of chemotherapy observed in this case suggests that this approach might be reasonable to consider in the rare instance that a malignant glomus tumor presents with high-grade pathology and locally advanced presentation.

## Conclusions

In conclusion, we describe a rare case of a high-grade, locally advanced malignant glomus tumor arising from the liver. The case is notable for its favorable response to neoadjuvant chemotherapy, which to our knowledge has not previously been described in this disease entity. This case additionally highlights the utility of next-generation sequencing as a diagnostic aid for ultra-rare malignancies with unusual presentation.
